# Predicting the Course of Immunoglobulin A Nephropathy Using Urinary Soluble Cluster of Differentiation 163 as a Prognostic Biomarker

**DOI:** 10.7759/cureus.101499

**Published:** 2026-01-13

**Authors:** S. K. Afsana Hossain, Muhammad Nazrul Islam, A. H. Hamid Ahmed, H. M. Mohiuddin Alamgir, Md. Saiful Ahammad Sarker, Mohammad Kamrul Hasan, Tashnia Tamanna Chowdhury, Mamun Chowdhury Raju, A. K. M. Shahidur Rahman, S. M. Remin Rafi, Md. Masudul Karim

**Affiliations:** 1 Nephrology, Directorate General of Health Services (DGHS), Dhaka, BGD; 2 Nephrology, Bangladesh Medical University (BMU), Dhaka, BGD; 3 Nephrology, East West Medical College and Hospital, Dhaka, BGD

**Keywords:** biomarker, disease progression, iga nephropathy, response status, urinary scd163

## Abstract

Background: Immunoglobulin A nephropathy (IgAN) is the most prevalent primary glomerulonephritis (GN) worldwide and is often underdiagnosed because of its asymptomatic presentation. Proteinuria-based assessment in monitoring disease progression is limited. Early renal biopsy may fail to predict disease severity. So, the development of a non-invasive biomarker can predict early disease progression.

Objective: To evaluate urinary soluble cluster differentiation 163 (u-sCD163) in predicting short-term treatment response and its correlation with histologic activity in biopsy-proven IgAN.

Methodology: This prospective observational study was carried out at the Department of Nephrology, Bangladesh Medical University (BMU), Dhaka, Bangladesh. A total of 55 patients with biopsy-proven primary IgAN were enrolled. Urine routine microscopic examination (R/M/E), 24-hour urinary total protein (UTP), and serum creatinine were measured at baseline, and at each follow-up (every three-month interval, up to six months). u-sCD163 was measured at baseline and at the sixth month. Patients were managed according to the KDIGO, 2021guideline and followed up in the third and sixthmonths.

Results: Mean age of the study patients was 31.3 ± 5.61 years. The sex distribution highlights a male predominance, as the male-to-female ratio was 1.7:1. Baseline u-sCD163 was 3.78 ± 0.96 ng/mg in the partial response group and 7.82 ± 4.00 ng/mg in the no-response group (*P *< 0.001). The receiver operating characteristic (ROC) curve analysis of urinary sCD163 level for predicting no response at the third month showed an optimal cutoff value of 4.80, providing a sensitivity of 76.7% and specificity of 83.3%. At six months, urinary sCD163 levels were highest in the no-response group (7.22 ± 3.41 ng/mg, *P *< 0.001). u-sCD163 was markedly elevated in patients with endocapillary hypercellularity and crescents (*P *< 0.05).

Conclusions: u-sCD163 may be a potential biomarker to predict the short-term treatment response status and histologic activity in biopsy-proven IgAN. Elevated biomarkers at baseline were associated with more proteinuria and risk of response failure.

## Introduction

Immunoglobulin A (IgA) nephropathy (IgAN) is an autoimmune kidney disorder characterized by the deposition of IgA antibodies in the renal glomeruli, which ultimately leads to inflammation and damage. A significant percentage (20%-40%) of patients with IgAN develop end-stage renal disease (ESRD) within 20 years of diagnosis [1]. The global incidence of IgAN is estimated at about 2.5 cases per 100,000 people per year, and the prevalence of IgAN varies globally, with higher rates observed in East Asia, particularly Japan and Korea [2]. IgAN is one of the commonest primary glomerulonephritides worldwide that can manifest clinically in a variety of ways, such as asymptomatic microscopic hematuria along with varying degrees of proteinuria; synpharyngitic macroscopic hematuria; rapidly progressive glomerulonephritis (RPGN), and nephrotic syndrome (NS) [3]. It's crucial to remember that the illness can develop gradually and that some people might not exhibit any symptoms until serious kidney damage has taken place [3]. IgAN is explained by a multi-hit hypothesis of pathogenesis: Hit 1, excessive production of galactose-deficient IgA1; Hit 2, generation of autoantibodies against galactose-deficient IgA1; Hit 3, formation of immune complexes consisting of IgG autoantibodies bound to galactose-deficient IgA1; and Hit 4, deposition of these immune complexes in the glomeruli, which activates mesangial cells and leads to the production of inflammatory mediators such as interleukin-6 and platelet-derived growth factor, as well as complement activation, resulting in monocyte infiltration and glomerular injury [4]. Macrophage infiltration, particularly CD163-positive (CD163⁺) regulatory macrophages, plays a pivotal role in the development of IgAN [5]. These cells, distinguished by the CD163 surface marker, contribute to kidney tissue injury, glomerular damage, and increased disease severity in affected individuals [5]. In the kidney, the quantity of CD163+ macrophages correlates with the severity of disease, affecting proteinuria, serum albumin levels, and the glomerular filtration rate (GFR) [5]. The CD163 is a transmembrane protein having a molecular weight of 130 kDa. These macrophages play a role in acute tubular damage, glomerular lesions, and active crescent formation in IgAN [5].

It was reported that urinary soluble CD163 (u-sCD163) levels serve as a non-invasive biomarker in IgAN, indicating disease severity and response status [6]. Elevated u-sCD163 levels are associated with increased proteinuria, renal dysfunction, and severe histological lesions. Additionally, higher u-sCD163 levels are linked to a higher risk of response failure in patients with IgAN [6]. Proteinuria is a crucial indicator of renal function in patients with IgAN, with persistent proteinuria (>0.75-1 g/24h) after 90 days of optimal supportive treatment indicating a high risk of progression in IgAN [7]. It is still difficult to differentiate between proteinuria caused by chronic sclerotic glomerular damage and acute glomerular inflammatory lesions such as cellular crescents in IgAN [8]. The Oxford MEST-C grading system from kidney biopsies helps predict renal outcomes in IgAN [3]. The VALIGA trial confirmed that specific grades like M1, S1, and T1/2 are linked to poor outcomes, emphasizing the role of histology in prognosis [9]. Early-stage renal biopsies sometimes provide inconclusive results; therefore, the need for new noninvasive biomarkers to evaluate disease activity in real-time is highlighted for better risk assessment [8]. Due to limitations in kidney biopsy and urinalysis, alternative noninvasive assessment techniques based on reliable biomarkers are necessary for early diagnosis and efficient monitoring of IgAN progression. This study was intended to evaluate the link between u-sCD163 and short-term treatment response and disease status in patients with IgAN.

## Materials and methods

It was a prospective observational study carried out at the Department of Nephrology, Bangladesh Medical University (BMU), Dhaka, Bangladesh, from June 2023 to December 2024. Initially, a total of 60 patients with biopsy-proven IgAN were enrolled in this study by a purposive sampling technique. Of them, 5 patients dropped out (3 patients did not come for follow-up, 2 patients required immunosuppressive therapy other than the Manno regimen-so discontinued from the study). Finally total of 55 biopsy-proven patients with IgAN were evaluated. All study patients were selected following the selection criteria. Patients with biopsy-proven IgAN who were at least 18 years old or above were included. Patients with an estimated glomerular filtration rate (eGFR) <15 mL/min, secondary mesangial IgA deposition due to hepatitis-related glomerulonephritis (GN), rheumatoid arthritis, systemic lupus erythematosus, Henoch-Schönlein purpura, sepsis, or pregnancy were excluded. All selection criteria were assessed using prior medical history, clinical examination findings, review of past medical records, and laboratory investigations.

Study procedure

After obtaining Institutional Review Board (IRB) approval, 55 study participants were informed about the natural history and pathophysiology of IgAN, relevant investigations, current treatment options, outcomes, and potential consequences of the disease. Written informed consent was then obtained from each participant. Age, sex, body mass index (BMI), and blood pressure (BP) were recorded for each study participant. Hypertension (HTN) was defined as a systolic blood pressure (SBP) ≥140 mmHg, a diastolic blood pressure (DBP) ≥90 mmHg, or the use of antihypertensive medication. Laboratory investigations included routine urine microscopy (R/M/E), 24-hour urinary total protein (24h UTP), u-sCD163, serum creatinine, eGFR, hemoglobin (Hb), and serum albumin levels.

Sample collection, processing, and analysis

Urine and blood samples were collected with aseptic precautions. A clean-catch midstream urine sample was collected from all patients and stored at −80 °C until measurement of u-sCD163. u-sCD163 was quantified using a commercially available enzyme-linked immunosorbent assay (ELISA) kit according to the manufacturer’s protocol (Elabscience; sensitivity 0.94 ng/mL; intra-assay coefficient of variation <10%). 24h UTP was measured using a turbidimetric method. Blood samples were collected by venipuncture, allowed to clot, and the serum was separated by centrifugation at room temperature. Serum creatinine was measured by the colorimetric method. The photometric method was used to measure serum albumin level. eGFR was calculated by using the Modification of Diet in Renal Disease (MDRD) formula [7]. Treatment was administered according to the KDIGO 2021 guidelines [7].

**Figure 1:**
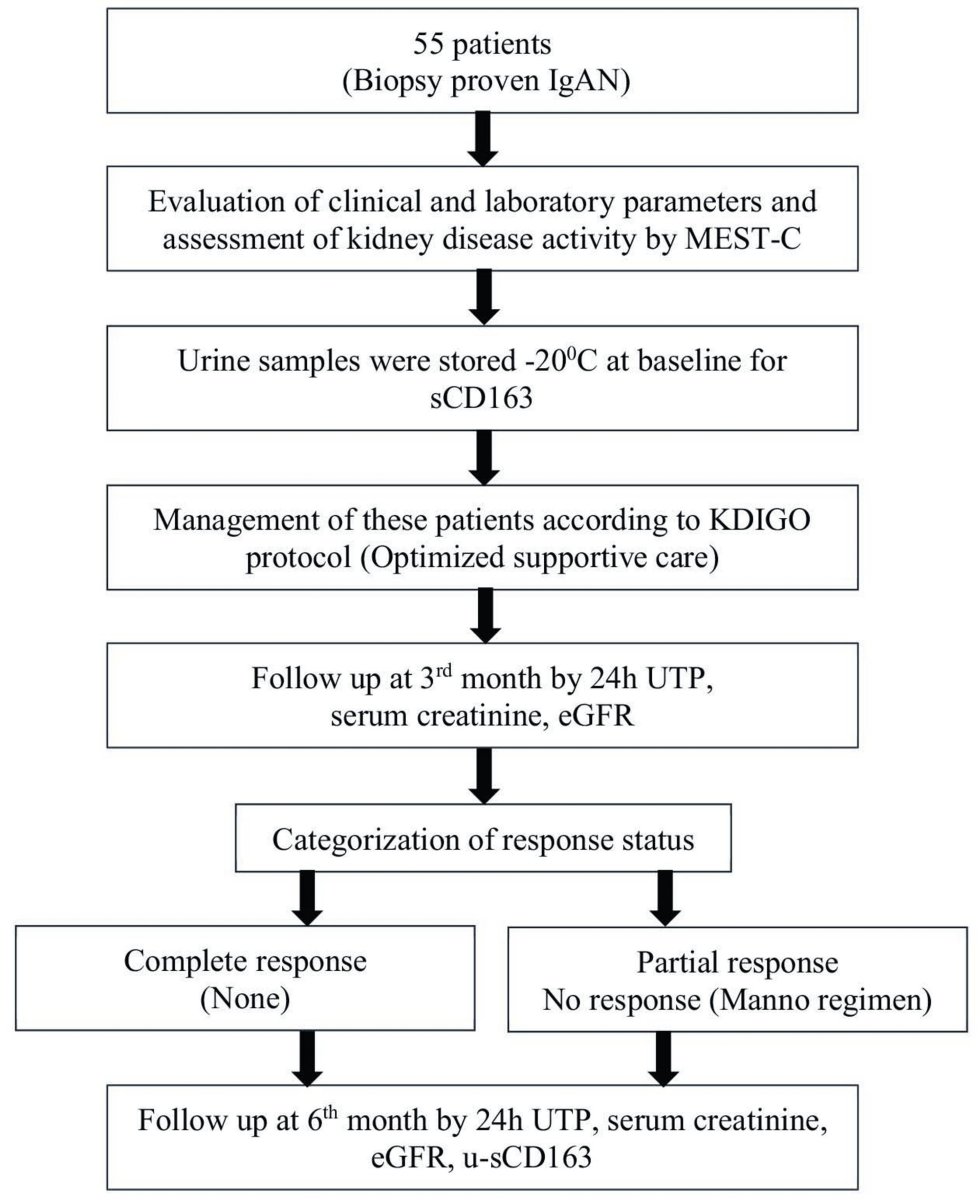
Flowchart displaying the study procedure, management, and follow-up schedule. Image credit: S. K. Afsana Hossain. IgAN, Immunoglobulin A nephropathy; MEST-C, mesangial hypercellularity, endocapillary hypercellularity, segmental sclerosis, tubular atrophy/interstitial fibrosis, and crescents; sCD163, soluble cluster differentiation 163; KDIGO, Kidney Disease Improving Global Outcomes; 24h UTP, 24-hour urinary total protein; eGFR, estimated glomerular filtration rate; u-sCD163, urinary soluble cluster differentiation 163

The study procedure, management, and follow-up schedule are depicted in Figure 1. 

Renal histology graded by the Oxford MEST-C score

Mesangial hypercellularity was recorded based on the number of cells per mesangial area and categorized as M0 or M1. Endocapillary hypercellularity was scored as absent (E0) or present (E1). Segmental sclerosis was classified as absent (S0) or present (S1). Interstitial fibrosis and tubular atrophy were graded according to the proportion of tubulointerstitial involvement as T0 (≤25%), T1 (26%-50%), or T2 (>50%). Crescent formation was scored as C0 when absent, C1 when present in <25% of glomeruli, and C2 when present in ≥25% of glomeruli [3].

Operational definitions

Optimized Supportive Care

Optimized supportive care for IgAN focused on slowing disease progression, managing symptoms, and improving quality of life. Blood pressure was maintained at or below 130/80 mmHg using angiotensin-converting enzyme (ACE) inhibitors or angiotensin II receptor blocker (ARB), with calcium channel blocker (CCB) added when required. Proteinuria was managed primarily with ARBs, even in patients with normal or near-normal blood pressure. Lifestyle modifications, including a low-sodium diet, maintenance of ideal body weight, and cessation of smoking, were advised for all patients [10].

Manno Regimen

Oral prednisolone 1 mg/kg (maximum 75 mg/day) for two months; tapering at 0.2 mg/kg per month; total treatment duration six months [7].

Treatment responses are illustrated in Table 1 [6].

**Table 1:** Treatment responses. eGFR, estimated glomerular filtration rate; ESRD, end-stage renal disease

Response category	Definition
Complete response	Proteinuria <0.3 g/24h with no worsening of kidney function (<30% reduction in eGFR from baseline)
Partial response	Proteinuria 0.3 to ≤1.0 g/24h with no worsening of kidney function (<30% reduction in eGFR from baseline)
No response	Proteinuria >1.0 g/24h or increase from baseline with worsening kidney function (>30% reduction in eGFR) or progression to ESRD

Data analysis

Statistical analyses were performed using the Windows-based Statistical Package for the Social Sciences (SPSS), version 26 (IBM Corp., Armonk, NY). The Mann-Whitney U test and the Kruskal-Wallis test were used for continuous variables. Spearman’s correlation analysis was used to assess the relationship between u-sCD163 and proteinuria as well as serum creatinine. Receiver operating characteristic (ROC) curve analysis was performed to determine the sensitivity and specificity of urinary u-sCD163 in predicting histopathology and treatment response. A *P*-value <0.05 was considered statistically significant.

## Results

This study was conducted on 55 biopsy-proven primary patients with IgAN to predict the course of the disease by u-sCD163 as a biomarker. Table 1 shows that the study population consisted predominantly of younger individuals, with the majority (24, 43.6%) being under 30 years of age, followed by 20 (36.4%) aged 30-39 years, and only 11 (20.0%) aged 40-50 years. The sex distribution highlights a male predominance, with 35 (63.6%) males and 20 (36.4%) females, resulting in a male-to-female ratio of 1.7:1. The largest group of participants (25, 45.5%) fell into the overweight category (body mass index (BMI) 25-29.9 kg/m²), followed by 17 (30.9%) in the normal weight range (BMI 18.5-24.99 kg/m²) and 13 (23.6%) classified as obese (BMI >30 kg/m²). The most common presentation was sub-nephrotic proteinuria with hematuria, observed in 33 (60.0%) patients, followed by NS in 16 (29.1%) patients. HTN was present in 29 patients (52.7%), and renal impairment at presentation was documented in 31 patients (56.4%). Analysis of the histopathological findings showed that mesangial hypercellularity was the most common feature, present in 53 patients (96.4%). Endocapillary hypercellularity was observed in 24 patients (43.6%), segmental sclerosis in 32 patients (58.2%), mild interstitial fibrosis/tubular atrophy (T1) in 27 patients (49.1%), and crescents in 15 patients (27.3%). All patients received optimized supportive care; 41 patients (74.5%) were treated with the Manno regimen, while the remaining 14 (25.5%) were managed conservatively (Table 2).

**Table 2:** Basic data of the study patients (N = 55). SD, standard deviation; BMI, body mass index; UTP, urinary total protein; BP, blood pressure

Variables	*n* (%)
Age (years)	
<30	24 (43.6)
30-39	20 (36.4)
40-50	11 (20.0)
Mean ± SD	31.3 ± 5.61
Sex	
Male	35 (63.6)
Female	20 (36.4)
BMI categories	
Normal weight	17 (30.9)
Overweight	25 (45.5)
Obese	13 (23.6)
Clinical presentation	
Sub-nephrotic proteinuria with hematuria	33 (60)
Nephrotic syndrome (UTP >3.5 g/24h)	16 (29.1)
Hypertension (BP ≥140/90 mmHg)	29 (52.7)
Renal impairment at presentation (serum creatinine >1.2 mg/dL)	31 (56.4)
Histopathological findings	
Mesangial hypercellularity	53 (96.4)
Endocapillary hypercellularity	24 (43.6)
Segmental sclerosis	32 (58.2)
Interstitial fibrosis/tubular atrophy	27 (49.1)
Crescents	15 (27.3)
Treatment protocol	
Optimized supportive care	55 (100)
Specific treatment	
Manno regimen	41 (74.5)
Conservative management	14 (25.5)

Table 3 presents the baseline laboratory parameters of the study patients. Proteinuria was most commonly graded as ++ in 27 patients (49.1%), followed by +++ in 15 patients (27.3%) and ++++ in 11 patients (20.0%). More than 5 RBCs/HPF were found in 26 (47.3%) patients, while 17 (30.9%) patients had WBCs >5/HPF. A high 24h UTP level (>3.5 g/24h) was noted in 22 (41.8%) patients, serum creatinine was high (>1.2 mg/dL) in 31 (56.4%) study patients, and 26 (47.3%) patients had eGFR values below 60 mL/minute/1.73 m².

**Table 3:** Baseline laboratory parameters of study patients (N = 55). Urine R/M/E, urine routine microscopic examination; RBC, red blood cell; HPF, high-power field; WBC, white blood cell; 24h UTP, 24-hour urinary total protein; eGFR, estimated glomerular filtration rate

Parameters	Frequency (*n*)	Percentage (%)
Urine R/M/E		
Proteinuria		
+	2	3.6
++	27	49.1
+++	15	27.3
++++	11	20.0
RBC (HPF)		
0-5	29	52.7
>5	26	47.3
WBC (HPF)		
<5	37	69.1
>5	17	30.9
24h UTP (g/24h)		
<3.5	33	60.0
>3.5	22	41.8
Serum albumin (g/L)		
<35	18	32.7
>35	37	67.2
Serum creatinine (mg/dL)		
<1.2	24	43.6
>1.2	31	56.4
eGFR (mL/minute/1.73 m^2^)		
15-30	14	25.5
30-45	7	12.7
45-60	5	9.1
60-90	20	36.4
>90	9	16.4

In this study, baseline u-sCD163 level was mildly higher in the M1 group than the M0 group (6.99 ± 3.99 ng/mg versus 5.49 ± 1.81 ng/mg, *P* = 0.600). However, baseline u-sCD163 level was significantly higher in the E1 group compared to the E0 group (8.68 ± 4.70 ng/mg versus 5.59 ± 2.56 ng/mg, *P *= 0.003). The baseline u-sCD163 level was slightly higher in the S1 group compared to the S0 group (6.98 ± 3.19 ng/mg versus 6.27 ± 2.31 ng/mg, *P* = 0.212). The baseline u-sCD163 level was relatively higher in the T1 group compared to the T0 group (7.72 ± 3.44 ng/mg versus 6.09 ± 3.15 ng/mg, *P* = 0.064). The baseline u-sCD163 level was significantly higher in the C1 group compared to the C0 group (9.80 ± 4.77 ng/mg versus 5.87 ± 2.98 ng/mg, *P* = 0.001) (Table 4). 

**Table 4:** Association of baseline urinary soluble CD163 levels with Oxford MEST-C renal histopathology grades (N = 55). *P*-value was obtained by the Mann-Whitney test. *Significant. MEST-C, mesangial hypercellularity, endocapillary hypercellularity, segmental sclerosis, tubular atrophy/interstitial fibrosis, and crescents; SD, standard deviation

Oxford MEST-C	Grades	n	Urinary soluble CD163 (ng/mg) (Mean ± SD)	*P*-value
Mesangial hypercellularity (M)	M0	2	5.49 ± 1.81	0.600
	M1	53	4.20 ± 6.80	
Endocapillary hypercellularity (E)	E0	31	5.59 ± 2.56	0.003*
	E1	24	8.68 ± 4.70	
Segmental sclerosis (S)	S0	23	6.27 ± 2.31	0.212
	S1	32	6.98 ± 3.19	
Interstitial fibrosis/tubular atrophy (T)	T0	27	6.09 ± 3.15	0.064
	T1	28	7.72 ± 3.44	
Crescents (C)	C0	40	5.87 ± 2.98	0.001*
	C1	15	9.80 ± 4.77	

Among the study patients, 43 (78.2%) showed no response, while 12 (21.8%) exhibited a partial response at three months. The baseline u-sCD163 level was 3.78 ± 0.96 ng/mg in the partial response group and 7.82 ± 4.00 ng/mg in the no-response group (*P* < 0.001) (Table 5).

**Table 5:** Association of baseline urinary soluble CD163 with response status at three months (N = 55). *P*-value was obtained by the Mann-Whitney test. *Significant. SD, standard deviation

Parameter	Partial response (*n* = 12) (Mean ± SD)	No response (n = 43) (Mean ± SD)	*P*-value
Baseline urinary soluble CD163 (ng/mg)	3.78 ± 0.96	7.82 ± 4.00	<0.001*

**Figure 2:**
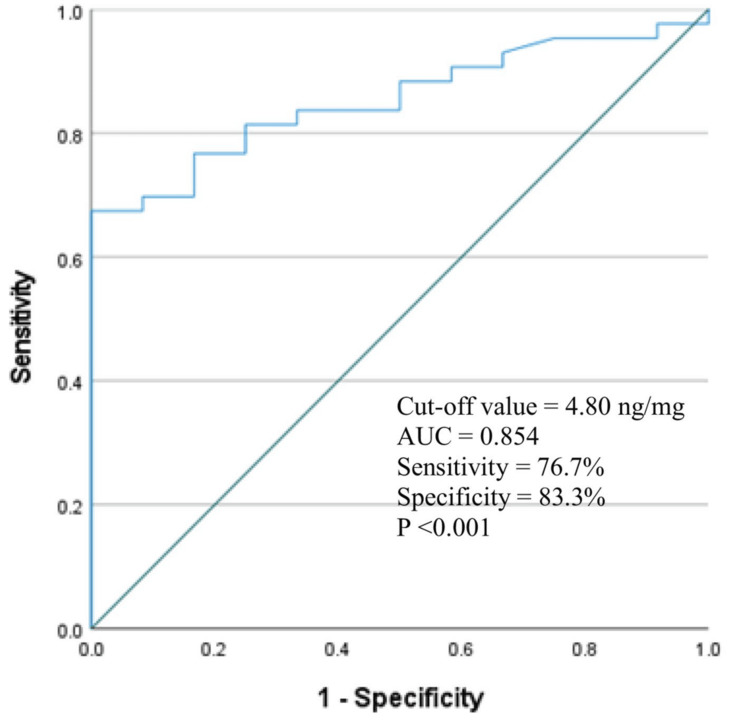
ROC curve analysis demonstrating the optimal cutoff of baseline urinary soluble CD163 levels for predicting no response at three months in patients with IgA nephropathy. Diagonal segments are produced by ties. AUC, area under the curve; ROC, receiver operating characteristic

Figure 2 presents the ROC curve analysis of u-sCD163 levels for predicting non-response in patients with IgAN. The analysis demonstrated a good predictive ability, with an AUC of 0.854 (95% CI: 0.755-0.952, *P* < 0.001). The optimal cutoff value for u-sCD163 was 4.80 ng/mg, yielding a sensitivity of 76.7% and a specificity of 83.3% (Figure 2)

At six months (after three months of the Manno regimen), 37 (67.3%) had no response, 8 (14.5%) had a partial response, and 10 (18.2%) had a complete response. It was observed that the u-sCD163 level was lowest in the complete response group and highest in the no-response group (2.91 ± 1.03 ng/mg versus 7.22 ± 3.41 ng/mg, *P *< 0.001) (Table 6).

**Table 6:** Association of urinary soluble CD163 with response status at six months (N = 55). *P*-value obtained by the Kruskal-Wallis test. *Significant SD, standard deviation

Parameter	Response status at six months			*P*-value
	Complete response (*n* = 10) (Mean ± SD)	Partial response (*n* = 8) (Mean ± SD)	No response (*n* = 37) (Mean ± SD)	
Urinary soluble CD163 at six months (ng/mg)	2.91 ± 1.03	4.33 ± 1.54	7.22 ± 3.41	<0.001*

Table 7 shows the correlation of u-sCD163 with 24h UTP. The baseline urinary CD163 level was positively correlated with 24h UTP at baseline (ρ = 0.530, *P* = 0.002), third month (ρ = 0.646, *P *< 0.001), and sixth month (ρ = 0.629, *P *< 0.001).

**Table 7:** Correlation of baseline urinary soluble CD163 with 24-hour urinary total protein (UTP) and serum creatinine (N = 55).

Variables	Spearman’s ρ (Rho)	*P*-value
24h UTP		
Baseline 24h UTP	0.530	0.002
24h UTP at 3 months	0.646	<0.001
24h UTP at 6 months	0.629	<0.001
Serum creatinine		
Baseline serum creatinine	0.410	0.002
Serum creatinine at 3 months	0.470	<0.001
Serum creatinine at 6 months	0.488	<0.001

Regarding the correlation of u-sCD163 with serum creatinine, baseline u-sCD163 levels were positively correlated with serum creatinine at baseline (ρ = 0.410, *P* = 0.002), at three months (ρ = 0.470, *P* < 0.001), and at six months (ρ = 0.488, *P* < 0.001), indicating that higher u-sCD163 levels are associated with increased renal dysfunction (Table 7).

Table 8 shows that age had a significant positive correlation with u-sCD163 (ρ = 0.478, *P *< 0.001), suggesting that older patients had higher biomarker levels. BMI showed a weak, non-significant correlation with u-sCD163 (*P* > 0.05). HTN also had a significant positive correlation with u-sCD163 (ρ = 0.344, *P* = 0.010).

**Table 8:** Correlation of baseline u-sCD163 with clinical parameters (N = 55). BMI, body mass index; HTN, hypertension; u-sCD163, urinary soluble cluster differentiation 163

Clinical parameters	u-sCD163	
	Rho (ρ)	*P*-value
Age	0.478	0.001
BMI	0.232	0.088
HTN	0.344	0.010

## Discussion

Worldwide, IgAN is one of the most common primary glomerulonephritides. Deposition of IgA in the glomerular mesangium is the hallmark of IgAN, leading to inflammation and progressive renal damage. Patients with IgAN may exhibit a variety of symptoms, ranging from asymptomatic microscopic hematuria to macroscopic hematuria and/or proteinuria. The current method of diagnosing IgAN is a kidney biopsy, which is an invasive procedure. Due to the potential risks associated with renal biopsy, such as bleeding and other complications, there is a growing need for noninvasive biomarkers to evaluate disease severity in patients with IgAN. This study investigated the association between u-sCD163 levels and histological activity, as well as clinical and laboratory indicators, in a cohort of 55 patients with biopsy-proven IgAN. In this study, the demographic characteristics revealed a younger cohort, with the majority of participants (24, 43.6%) being around 30 years of age and a mean age of 31.3 ± 5.61 years. This age distribution suggests that IgAN is more prevalent in younger individuals, consistent with previous research reporting a mean age of 33.7 years, with an age range of 16 to 76 years [11]. Reflecting on the findings from previous studies, our research also found a male predominance, with 35 (63.6%) of participants being male and 20 (36.4%) being female, a male-to-female ratio of 1.7:1, aligning with the established trend of IgAN being more common in males [11].

Recent research has investigated the link between obesity and IgAN, but the findings remain inconsistent. Some research suggests that an increased BMI is closely linked to IgAN progression and an increased incidence of interstitial fibrosis [12], while Ouyang et al. proposed that a low BMI may be associated with disease progression [13]. In this study majority of participants (25, 45.5%) were overweight (BMI 25-29.9 kg/m²), highlighting the importance of considering BMI in both diagnosis and management of IgAN. This finding supports the notion that BMI could influence IgAN progression.

The baseline laboratory findings revealed important clinical parameters indicative of impaired renal function, as indicated by elevated serum creatinine (>1.2 mg/dL) in 31 (56.4%) patients and reduced eGFR in 17 (30.9%) cases, reflecting worsening renal function among the study cases.

The study highlighted the variability in clinical presentations of IgAN. Sub-nephrotic proteinuria with hematuria was observed in 33 patients (60%), followed by NS in 16 patients (29.1%), making it the most common presentation. It has been documented that NS is relatively uncommon in IgAN, occurring in only 5%-10% of patients [14]. However, the presence of NS in IgAN at onset can significantly affect the clinical presentation, management, and prognosis of the disease [14]. A previous study observed that 14.7% patients with IgAN presented with NS [15]. The NS group exhibited more pronounced pathological features, including endocapillary hypercellularity, tubular atrophy, interstitial fibrosis, and crescent formation, while showing fewer signs of segmental glomerulosclerosis, adhesion, and global sclerosis [15]. These findings support the idea that IgAN with NS represents a more severe and aggressive form of the disease, associated with a high risk of progression to ESRD. The presence of acute lesions such as crescents and endocapillary hypercellularity, alongside reduced eGFR and elevated serum creatinine levels, emphasizes the need for intense monitoring and more intensive treatment strategies for these patients [15].

In this study, at presentations, HTN was found in 29 (52.7%) patients, and renal impairment was observed in 31 (56.4%) patients. Interestingly, u-sCD163 was positively correlated with HTN, suggesting that a higher level of the marker was associated with a higher incidence of HTN, which may have implications for understanding the renal and cardiovascular complications in IgAN.

In this study, all patients were managed by optimized supportive care: of them, 41 (74.5%) were given the Manno regimen at the third month, while the remaining 14 (25.5%) patients did not fulfill the criteria for the Manno regimen.

Histopathological analysis showed that mesangial hypercellularity was in 53 (96.4%) patients, segmental sclerosis was in 32 (58.2%) patients, mild interstitial fibrosis/tubular atrophy was in 27 (49.1%) patients, endocapillary hypercellularity was in 24 (43.6%) patients, and crescents was in 15 (27.3%) patients, which align with findings from other studies [6,16]. u-sCD163 levels were significantly higher in patients with endocapillary hypercellularity (E1) and crescents (C1). However, the difference in soluble CD163 level was not statistically significant for mesangial hypercellularity (M1). In the study by Gong et al., u-sCD163 levels were significantly elevated in patients with mesangial hypercellularity, endocapillary hypercellularity, segmental glomerulosclerosis, tubulointerstitial damage, and renal crescent formation [6]. They also found strong correlations between u-sCD163 levels and histopathological scores like MEST-C, indicating its potential as a noninvasive biomarker for assessment of response status in IgAN [6].

Given the progressive nature of IgAN, we explored the association between inflammatory biomarkers and disease severity. Soluble urinary CD163 level was positively correlated with 24h UTP, further linking elevated CD163 levels to increased proteinuria, a sign of kidney damage and declining renal function. Previous studies have linked u-sCD163 to proteinuria in IgAN [6,17]. Gong et al. reported that patients with u-sCD163 >3.57 ng/mg Cr had significantly higher proteinuria (2.33 ± 1.36 g/24h) than those in the lowest tertile (0.75 ± 0.63 g/24h, *P *< 0.0001) [6]. Our findings align with this study, confirming the biomarker-proteinuria association. u-sCD163 level showed a strong positive correlation with serum creatinine at baseline, three months, and six months, indicating that higher levels of these markers are linked to worsening renal function, consistent with the findings of a related study [6]. Among the study population, patients in the partial response group had significantly lower CD163 levels compared to those with no response at both baseline and six months (*P *< 0.001). Likewise, those who achieved complete response had the lowest biomarker levels, while patients with no response consistently showed the highest levels throughout (*P *< 0.001).

In ROC curve analysis, u-sCD163 levels showed a moderate predictive ability of no response at the third month in patients with IgAN (AUC = 0.854, 95% CI: 0.755-0.952, *P *= 0.000). The optimal cutoff value for u-sCD163 was 4.80 ng/mg, with a sensitivity of 76.7% and a specificity of 83.3%. At six months (after three months of the Manno regimen), 37 patients (67.3%) showed no response, 8 patients (14.5%) had a partial response, and 10 patients (18.2%) had a complete response. It was observed that the u-sCD163 level was lowest in the complete response group and highest in the no-response group (2.91 ± 1.03 ng/mg versus 7.22 ± 3.41 ng/mg, *P *< 0.001).

A total of 41 (74.5%) of patients received treatment following the Manno regimen, reflecting the real-world application of the Manno trial’s conclusion that combination therapy with corticosteroids and ACE inhibitors effectively slows IgAN progression [18]. This connects directly to our findings, where a higher level of u-sCD163 was linked to worsening kidney function and proteinuria. Applying the Manno regimen in these patients may help reduce inflammation linked to elevated biomarker levels, potentially leading to improved kidney function. This study emphasizes the importance of monitoring this biomarker to gain deeper insight into treatment effectiveness and track disease progression more accurately.

The present investigation had several limitations. In this study, the purposive sampling technique was adopted. It was a single-center study with a relatively small sample size, limiting generalizability. Long-term renal outcome could not be evaluated. The urinary biomarker level was not correlated with the disease-specific treatment regimen. Moreover, glomerular CD163-positive macrophage infiltration was not observed in renal histopathology. Prospective multi-center studies with larger sample sizes and long-term follow-up are needed to confirm generalizability.

## Conclusions

u-sCD163 shows a potential biomarker to predict the short-term treatment response status and histologic activity in biopsy-proven IgA nephropathy. Elevated biomarker level at baseline is associated with more proteinuria and risk of response failure at the third month. It was observed that u-sCD163 was markedly elevated in patients with endocapillary hypercellularity and crescents. An elevated level of this marker correlates with more disease severity and response failure, making it valuable for early risk assessment and prognosis. Early detection of high-risk patients may be facilitated by integrating u-sCD163 estimation into clinical practice, enabling early interventions to slow disease progression. Future therapeutic strategies should explore targeted interventions based on biomarker levels to improve patient outcomes and reduce renal function decline. During the course of acute kidney injury (AKI) in IgAN, u-sCD163 could differentiate crescentic transformation from other causes of AKI.
